# Supplementary material for: Efficacy and Safety of a Personalized Vitamin D3 Loading Dose Followed by Daily 2000 IU in Colorectal Cancer Patients with Vitamin D Insufficiency: Interim Analysis of a Randomized Controlled Trial
Source: Nutrients. 2022 Oct 28;14(21):4546. doi: 10.3390/nu14214546 (PMC9658724; doi:10.3390/nu14214546)
Supplement: Supplementary file 1 [file nutrients-14-04546-s001.zip › nutrients-1983859-supplementary.pdf]

Supplemental Material

Efficacy and Safety of a Personalized Vitamin D_3 Loading Dose Followed by Daily 2000 IU in Colorectal Cancer Patients with Vitamin D Insufficiency: Interim Analysis of a Randomized Controlled Trial

Table of contents

Suppl. Figure S1. Box plots of changes in 25(OH)D levels from screening to end of rehabilitation (visit 1, end of loading dose, day 12-21) and from screening to end of the study (visit 2, end of maintenance dose, week 13-16) in the per-protocol analysis. 2

Suppl. Figure S2. Box plots of 25(OH)D levels during the course of the trial restricted to subjects with vitamin D deficiency (25(OH)D < 30 nmol/L) at screening (per-protocol analysis)........ 3

Suppl. Table S1. Exclusion criteria of the VICTORIA trial 4

Suppl. Table S2. Serum 25(OH)D levels at screening, visit 1 and visit 2 7

Suppl. Table S3. Changes of 25(OH)D levels from screening to visit 1 and from screening to visit 2 8

Suppl. Table S4. Prevalence of vitamin insufficiency during the course of the trial 9

Suppl. Table S5. Safety parameters of six patients who experienced hypercalciuria 10

Suppl. Table S6. Urinary calcium-to-creatinine ratio at screening, visit 1 and visit 2........ 11

Suppl. Table S7. Albumin-corrected serum calcium at screening, visit 1 and visit 2........ 12

Suppl. Table S8. Estimated glomerular filtration rate (eGFR) at screening, visit 1 and visit 2 13

Suppl. Table S9. Comparison of the distribution of loading doses hypothetically calculated with the equations of Jansen et al. and von Groningen et al. for the study participants of the VICTORIA trial........ 14

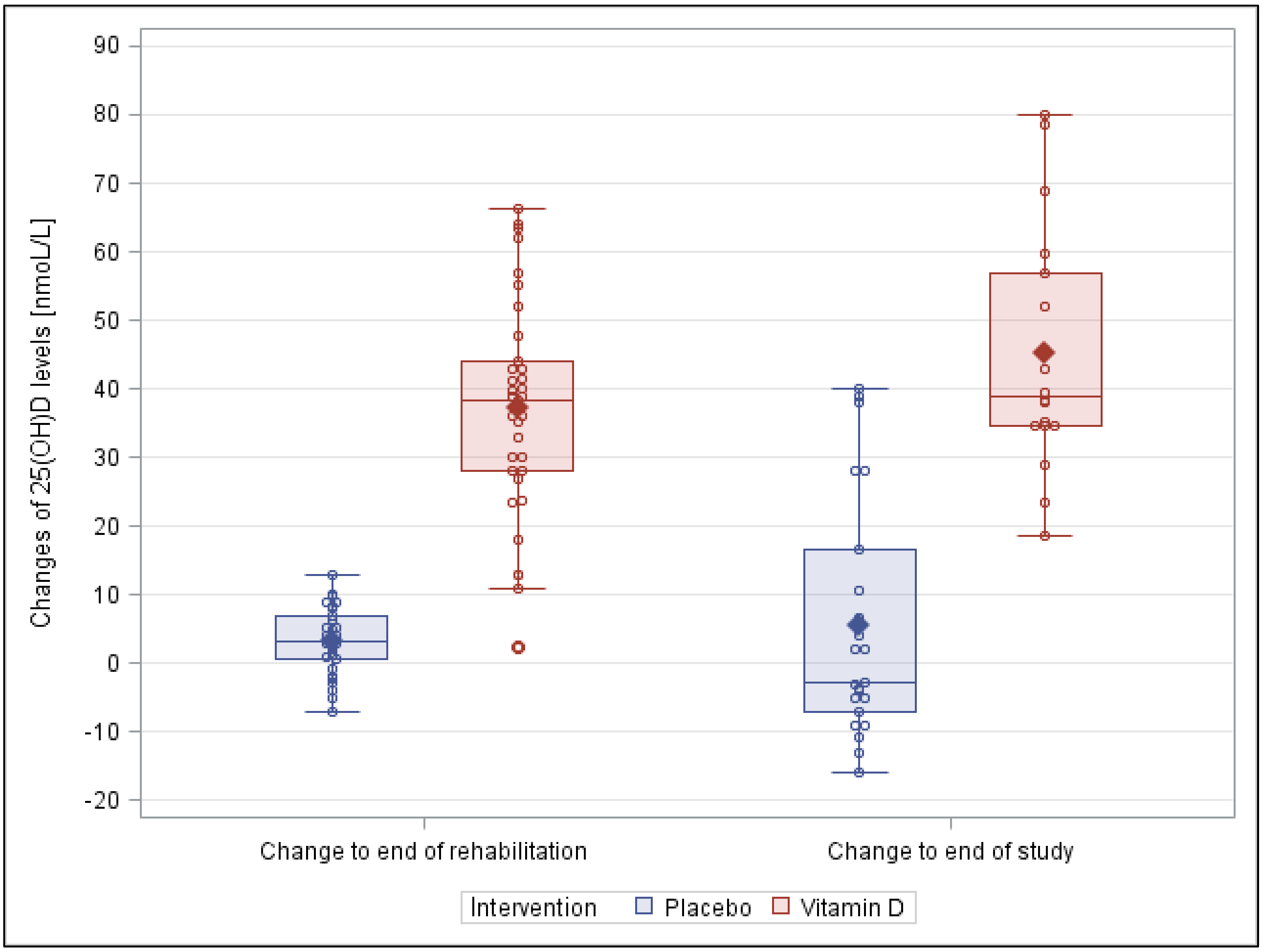

Suppl. Figure S1. Box plots of changes in 25(OH)D levels from screening to end of rehabilitation (visit 1, end of loading dose, day 12-21) and from screening to end of the study (visit 2, end of maintenance dose, week 13-16) in the per-protocol analysis.

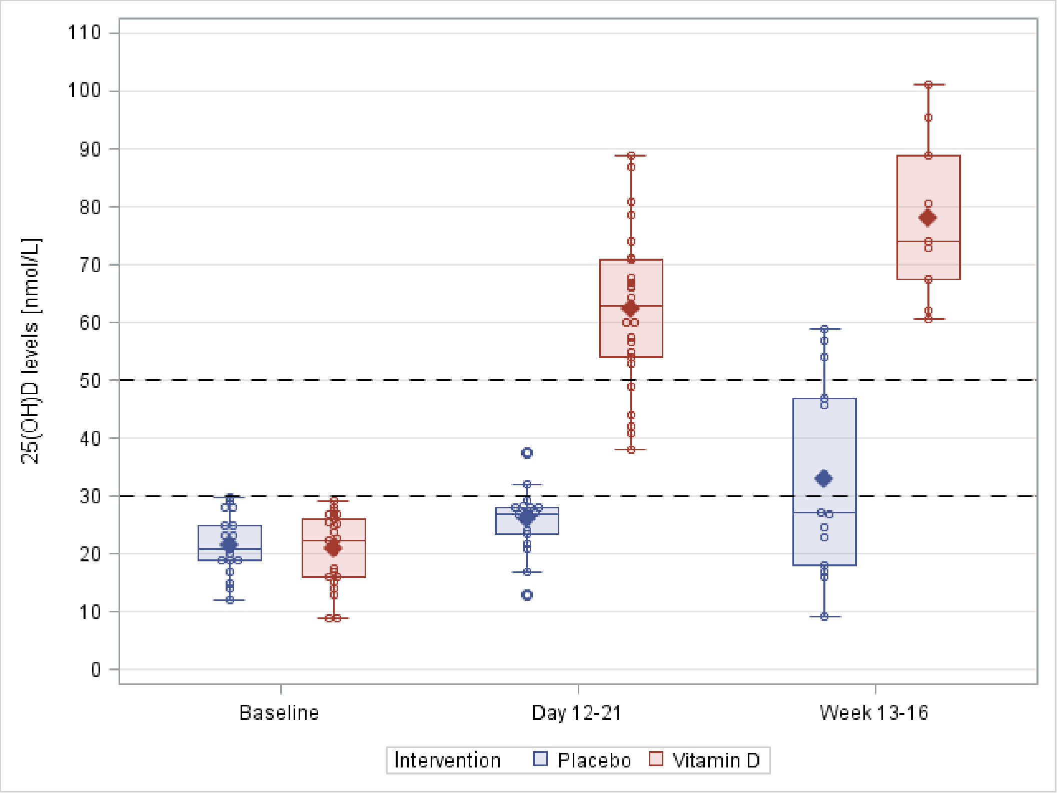

Suppl. Figure S2. Box plots of 25(OH)D levels during the course of the trial restricted to subjects with vitamin D deficiency (25(OH)D < 30 nmol/L) at screening (per-protocol analysis)

Suppl. Table S1. Exclusion criteria of the VICTORIA trial

Exclusion criterion	Ascertainment/ Operationalization	Justification
No vitamin D insufficiency or deficiency	Measurement	We do not expect an efficacy of a vitamin D_3 intervention for patients without vitamin D insufficiency or deficiency. The threshold for vitamin D insufficiency of the US American Institute of Medicine is 50 nmol/L.
Severe renal impairment	eGFR < 30 ml/min/1,73 m^2 calculated with Chronic Kidney Disease Epidemiology Collaboration (CKD-EPI) equation	Limited calcium and phosphate excretion; Special precautions for use of vitamin D_3 high-dose therapy according to Dekristol® 20 000 I.E. SmPC
Hypercalciuria	Random urine calcium ≥ 0.28 mg/mg creatinine (equals 0.79 mmol/mmol creatinine) [1]	Contraindication for vitamin D_3 high-dose therapy according to Dekristol® 20 000 I.E. (SmPC)
Hypercalcemia	Albumin-corrected serum calcium > 2.65 mmol/L [2]	Contraindication for vitamin D_3 high-dose therapy according to Dekristol® 20 000 I.E. (SmPC)
High-dose vitamin D_3 therapy	Vitamin D_3 daily ≥ 2,000 IU, vitamin D_3 weekly ≥ 14,000 IU or similar dosing regimen leading to average exposure to vitamin D_3 of ≥ 2,000 IU per day<br>Interview with the patient; Medical records;	Therapy would need to be stopped for trial participation.
Therapy with vitamin D analogs	Vitamin D_2 (Ergocalciferol), Dihydrotachysterol, Alfacalcidol, Calcitriol, or Calcifediol)	Therapy would need to be stopped for trial participation.
Topical therapy with vitamin D_3 or vitamin D analogs	Topical vitamin D_3 (e.g. Silikis®) or topical vitamin D analogs	Therapy would need to be stopped for trial participation.
Hypersensitivity to peanuts, soy, gelatin, lactose, maize starch or sucrose (ingredients in Dekristol® 20 000/1000 I.E.)	Interview with the patient	Contraindication for Dekristol® 20 000 I.E. or Dekristol® 1000 I.E. according to SmPC
Nephrolithiasis with symptoms in the last 12 months	Medical records	Condition can worsen because of increased serum calcium under

Exclusion criterion	Ascertainment/ Operationalization	Justification
		vitamin D_3 therapy. Contraindication for Dekristol® 1 000 I.E. according to SmPC
Pseudohypoparathyreodism	Medical records	Contraindication for vitamin D_3 high-dose therapy according to Dekristol® 20 000 I.E. SmPC; Risk of vitamin D_3 overdose
Sarcoidosis	Medical records	Increased production of the active form of vitamin D (1,25(OH)$_2$-vitamin D). Special precautions for use of vitamin D_3 high-dose therapy according to Dekristol® 20 000 I.E. SmPC
Therapy with cardiac glycosides	Medical records	Increased susceptibility to high calcium levels leads to enhanced risk of adverse effects from cardiac glycosides according to Dekristol® 20 000 I.E. SmPC
Therapy with high-dose calcium supplements	> 1000 mg calcium daily<br>Interview with the patient; Medical records	Simultaneous therapy with vitamin D_3 and high-dose calcium might increase the risk of stroke [3,4]
Participation in another intervention trial	Interview with the patient	To avoid potential conflicts in trial protocols and to ensure the safety of the participants by avoiding potential drug-drug interactions.
Pregnancy, planned pregnancy in next 12 weeks, or lactation	Urine pregnancy test during the screening phase	To ensure the safety of the unborn/newborn child.
No use of adequate contraceptive measures in women of childbearing potential	Interview with the patient	To ensure the safety of the unborn/newborn child.

Abbreviations: SmPC, Summary of Product Characteristics

References

1. Tellioglu, A.; Basaran, S.; Guzel, R.; Seydaoglu, G. Efficacy and safety of high dose intramuscular or oral cholecalciferol in vitamin D deficient/insufficient elderly. *Maturitas* **2012**, *72*, 332-338, doi:10.1016/j.maturitas.2012.04.011.
2. Meng, Q.H.; Wagar, E.A. Laboratory approaches for the diagnosis and assessment of hypercalcemia. *Crit. Rev. Clin. Lab. Sci.* **2015**, *52*, 107-119, doi:10.3109/10408363.2014.970266.

3. Jenkins, D.J.A.; Spence, J.D.; Giovannucci, E.L.; Kim, Y.-i.; Josse, R.; Vieth, R.; Blanco Mejia, S.; Viguiliouk, E.; Nishi, S.; Sahye-Pudaruth, S., et al. Supplemental Vitamins and Minerals for CVD Prevention and Treatment. *Journal of the American College of Cardiology* **2018**, *71*, 2570-2584, doi:https://doi.org/10.1016/j.jacc.2018.04.020.
4. Khan, S.U.; Khan, M.U.; Riaz, H.; Valavoor, S.; Zhao, D.; Vaughan, L.; Okunrintemi, V.; Riaz, I.B.; Khan, M.S.; Kaluski, E., et al. Effects of Nutritional Supplements and Dietary Interventions on Cardiovascular Outcomes: An Umbrella Review and Evidence Map. *Ann Intern Med* **2019**, *171*, 190-198, doi:10.7326/M19-0341.

Suppl. Table S2. Serum 25(OH)D levels at screening, visit 1 and visit 2

Intervention	ITT/ PP	N	25(OH)D (nmoL/L) Median	Mean (95%CI)	SD	Min	P25	P75	Max	P [a]
**Screening; day -8 to 0**										
No	ITT	33	28.0	28.4 (24.3 - 32.5)	11.6	12.0	20.0	32.5	61.0 [b]	0.360
Yes	ITT	37	26.0	26.0 (22.8 - 29.3)	9.7	9.0	20.4	31.3	48.5	
No	PP	29	25.0	27.6 (23.6 - 31.6)	10.5	12.0	20.0	32.5	48.0	0.501
Yes	PP	35	25.5	25.9 (22.5 - 29.3)	9.9	9.0	17.4	32.8	48.5	
**Visit 1; end of loading dose; day 12 to 21**										
No	ITT	32	28.0	31.6 (27.3 - 35.8)	11.8	13.0	26.1	38.3	68.0	<0.0001
Yes	ITT	36	64.6	63.1 (58.3 - 67.9)	14.3	33.5	54.5	71.6	91.0	
No	PP	29	28.0	31.0 (27.2 - 34.7)	9.9	13.0	26.3	37.5	58.0	<0.0001
Yes	PP	35	64.2	63.1 (58.1 - 68.0)	14.5	33.5	54.0	72.0	91.0	
**Visit 2; end of maintenance dose; week 13 to 16**										
No	ITT	27	30.2	35.1 (28.5 - 41.8)	16.8	9.2	23.0	45.8	76.0	<0.0001
Yes	ITT	25	71.9	72.5 (66.3 - 78.5)	14.9	30.8	65.8	80.5	101.0	
No	PP	23	30.2	34.1 (27.1 - 41.1)	16.1	9.3	23.0	45.8	76.0	<0.0001
Yes	PP	18	72.4	75.5 (69.2 - 81.9)	12.8	52.8	67.4	87.0	101.0	

Abbreviations: 25(OH)D, 25-hydroxyvitamin D; ITT, intention-to-treat analysis; Max, maximum; Min, minimum; P25, 25th Percentile; P75, 75th Percentile; PP, per-protocol analysis; SD, standard deviation.

[a] Two-sample two-tailed t-test used to test on difference of the means of two groups. Statistically significant in interim analysis if $p < 0.04$. The p-value of the test was derived by the Satterthwaite method.

[b] One patient without vitamin D insufficiency and a 25(OH)D level of 61.0 nmol/L was falsely included. Without this falsely included study participant, the maximum 25(OH)D level would have been 48.0 nmol/L.

Suppl. Table S3. Changes of 25(OH)D levels from screening to visit 1 and from screening to visit 2

Intervention	ITT/ PP	N	25(OH)D (nmoL/L)							P [a]
			Median	Mean (95%CI)	SD	Min	P25	P75	Max	
**Change from screening to visit 1**										
No	ITT	32	3.1	3.3 (1.5 - 5.0)	4.8	-7.3	0.5	7.0	13.0	<0.0001
Yes	ITT	36	38.5	37.2 (32.0 - 42.5)	15.4	2.3	28.0	43.5	66.4	
No	PP	29	3.3	3.3 (1.4 - 5.2)	5.0	-7.3	0.5	7.0	13.0	<0.0001
Yes	PP	35	38.3	37.2 (31.8 - 42.5)	15.6	2.3	28.0	44.0	66.4	
**Change from screening to visit 2**										
No	ITT	27	-1.3	5.6 (-1.0 - 12.3)	16.7	-16.0	-6.4	16.5	40.0	<0.0001
Yes	ITT	25	38.3	43.3 (36.0 - 50.6)	17.8	10.3	34.0	57.0	80.0	
No	PP	23	-2.8	5.5 (-2.1 - 13.1)	17.5	-16.0	-7.0	16.5	40.0	<0.0001
Yes	PP	18	38.9	45.0 (36.2 - 53.8)	17.7	18.5	34.5	57.0	80.0	

Abbreviations: 25(OH)D, 25-hydroxyvitamin D; ITT, intention-to-treat analysis; Max, maximum; Min, minimum; P25, 25th Percentile; P75, 75th Percentile; PP, per-protocol analysis; SD, standard deviation.

[a] Two-sample two-tailed t-test used to test on difference of the means of two groups. Statistically significant in interim analysis if p < 0.04. The p-value of the test was derived by the Satterthwaite method.

Suppl. Table S4. Prevalence of vitamin insufficiency during the course of the trial

Intervention	ITT/ PP	N	Vitamin D insufficiency (25(OH)D ≤ 50 nmoL/L)		P [a]
			No N (%)	Yes N (%)	
**Screening; day -8 to 0**					
No	ITT	33	1[b] (3.2)	32 (97.0)	NA
Yes	ITT	37	0 (0.0)	37 (100.0)	
No	PP	29	0 (0.0)	29 (100.0)	NA
Yes	PP	35	0 (0.0)	35 (100.0)	
**Visit 1; end of loading dose; day 12 to 21**					
No	ITT	32	2 (6.3)	30 (93.8)	<0.0001
Yes	ITT	36	29 (80.6)	7 (19.4)	
No	PP	29	1 (3.5)	28 (96.6)	<0.0001
Yes	PP	35	28 (80.0)	7 (20.0)	
**Visit 2; end of maintenance dose; week 13 to 16**					
No	ITT	27	5 (18.5)	22 (81.5)	<0.0001
Yes	ITT	25	24 (96.0)	1[c] (4.0)	
No	PP	23	4 (17.4)	19 (82.6)	<0.0001
Yes	PP	18	18 (100.0)	0 (0.0)	

Abbreviations: 25(OH)D, 25-hydroxyvitamin D; ITT, intention-to-treat analysis; NA, not applicable; PP, per-protocol analysis.

[a] Fisher's exact test. Statistically significant in interim analysis if $p < 0.04$.

[b] One patient without vitamin D insufficiency was falsely included.

[c] Treatment discontinuation after visit 1 due to hypercalciuria.

Suppl. Table S5. Safety parameters of six patients who experienced hypercalciuria

Arbitrary patient no.	Treatment arm	25(OH)D [nmol/L]	Albumin-corrected serum calcium [mmol/L]	Urinary calcium-to-creatinine ratio [mmol/mmol]	eGFR [ml/min/1,73 m^2]
**Screening visit; day -8 to 0**					
1	Placebo	17.0	2.5	0.6	88.1
2	Vitamin D	20.4	2.3	0.6	98.5
3	Vitamin D	13.0	2.2	0.4	102.9
4	Vitamin D	16.0	2.5	0.7	82.9
5	Vitamin D	25.0	2.5	0.8	94.3
6	Vitamin D	27.0	2.3	0.1	97.7
**Visit 1; end of loading dose; day 12 to 21**					
1	Placebo	13.0	2.4	0.8	96.7
2	Vitamin D	56.5	2.2	0.9	111.6
3	Vitamin D	49.0	2.3	0.9	101.1
4	Vitamin D	60.0	2.4	1.1	82.1
5	Vitamin D	89.0	2.4	1.4	95.8
6	Vitamin D	54.0	2.3	0.9	97.2
**Visit 2; end of maintenance dose; week 13 to 16**					
1	Placebo	40.0	2.4	0.6	88.1
2	Vitamin D	30.8	2.3	0.4	105.3
3	Vitamin D	-	-	-	-
4	Vitamin D	76.0	2.3	0.3	85.4
5	Vitamin D	-	-	-	-
6	Vitamin D	57.0	2.3	0.4	96.7

Abbreviations: 25(OH)D, 25-hydroxyvitamin D
Note: Hyphen instead of a numeric value means that value is missing.

Suppl. Table S6. Urinary calcium-to-creatinine ratio at screening, visit 1 and visit 2

Intervention	ITT/ PP	N	Urinary calcium-to-creatinine ratio [mmol/mmol]							p[a]
			Median	Mean (95%CI)	SD	Min	P25	P75	Max	
**Screening; day -8 to 0**										
Placebo	ITT	33	0.3	0.3 (0.2-0.4)	0.2	0.05	0.2	0.5	0.7	0.814
Vitamin D	ITT	37	0.2	0.3 (0.2-0.4)	0.2	0.02	0.1	0.4	0.8	
Placebo	PP	29	0.3	0.3 (0.2-0.4)	0.2	0.05	0.2	0.5	0.7	0.737
Vitamin D	PP	35	0.2	0.3 (0.2-0.4)	0.2	0.02	0.1	0.5	0.8	
**Visit 1; end of loading dose; day 12 to 21**										
Placebo	ITT	32	0.3	0.3 (0.2-0.4)	0.2	0.03	0.1	0.4	0.8	0.112
Vitamin D	ITT	36	0.3	0.4 (0.3-0.5)	0.3	0.07	0.2	0.6	1.4	
Placebo	PP	29	0.3	0.3 (0.2-0.4)	0.2	0.03	0.1	0.4	0.8	0.152
Vitamin D	PP	35	0.3	0.4 (0.3-0.5)	0.3	0.07	0.2	0.6	1.4	
**Visit 2; end of maintenance dose; week 13 to 16**										
Placebo	ITT	26	0.2	0.2 (0.2-0.3)	0.2	0.02	0.1	0.3	0.7	0.946
Vitamin D	ITT	25	0.2	0.2 (0.2-0.3)	0.1	0.04	0.2	0.4	0.5	
Placebo	PP	22	0.2	0.2 (0.2-0.3)	0.2	0.02	0.1	0.3	0.7	0.618
Vitamin D	PP	18	0.2	0.2 (0.2-0.3)	0.1	0.04	0.1	0.3	0.5	

Abbreviations: ITT, intention-to-treat analysis; Max, maximum; Min, minimum; P25, 25th Percentile; P75, 75th Percentile; PP, per-protocol analysis; SD, standard deviation.

[a] Two-sample two-tailed t-test used to test on difference of the means of two groups. Statistically significant in interim analysis if $p < 0.04$. The p-value of the test was derived by the Satterthwaite method.

Suppl. Table S7. Albumin-corrected serum calcium at screening, visit 1 and visit 2

Intervention	ITT/ PP	N	Albumin-corrected serum calcium [mmol/L]							p[a]
			Median	Mean (95%CI)	SD	Min	P25	P75	Max	
**Screening; day -8 to 0**										
Placebo	ITT	33	2.3	2.3 (2.3-2.3)	0.1	2.1	2.2	2.4	2.5	0.939
Vitamin D	ITT	37	2.3	2.3 (2.3-2.3)	0.1	2.1	2.2	2.3	2.5	
Placebo	PP	29	2.3	2.3 (2.3-2.3)	0.1	2.1	2.2	2.4	2.5	0.934
Vitamin D	PP	35	2.3	2.3 (2.3-2.3)	0.1	2.2	2.2	2.3	2.5	
**Visit 1; end of loading dose; day 12 to 21**										
Placebo	ITT	32	2.3	2.3 (2.3-2.3)	0.1	2.2	2.2	2.4	2.5	0.940
Vitamin D	ITT	36	2.3	2.3 (2.3-2.3)	0.1	1.9	2.2	2.4	2.4	
Placebo	PP	29	2.3	2.3 (2.3-2.3)	0.1	2.2	2.3	2.4	2.5	0.965
Vitamin D	PP	35	2.3	2.3 (2.3-2.3)	0.1	1.9	2.3	2.4	2.4	
**Visit 2; end of maintenance dose; week 13 to 16**										
Placebo	ITT	27	2.3	2.3 (2.2-2.3)	0.1	2.0	2.2	2.3	2.4	0.829
Vitamin D	ITT	25	2.3	2.3 (2.2-2.3)	0.1	2.1	2.2	2.3	2.4	
Placebo	PP	23	2.3	2.3 (2.2-2.3)	0.1	2.0	2.2	2.3	2.4	0.960
Vitamin D	PP	18	2.3	2.3 (2.2-2.3)	0.1	2.1	2.2	2.3	2.4	

Abbreviations: ITT, intention-to-treat analysis; Max, maximum; Min, minimum; P25, 25th Percentile; P75, 75th Percentile; PP, per-protocol analysis; SD, standard deviation.

[a] Two-sample two-tailed t-test used to test on difference of the means of two groups. Statistically significant in interim analysis if $p < 0.04$. The p-value of the test was derived by the Satterthwaite method.

Suppl. Table S8. Estimated glomerular filtration rate (eGFR) at screening, visit 1 and visit 2

Intervention	ITT/ PP	N	eGFR [ml/min/1,73 m^2]							p[a]
			Median	Mean (95%CI)	SD	Min	P25	P75	Max	
**Screening; day -8 to 0**										
Placebo	ITT	33	90.0	86.9 (81.7-92.1)	14.6	48.0	78.5	97.2	109.0	0.479
Vitamin D	ITT	37	93.0	89.5 (84.3-94.6)	15.4	55.5	78.5	98.5	120.6	
Placebo	PP	29	90.7	86.6 (80.7-92.5)	15.5	48.0	77.6	97.2	109.0	0.458
Vitamin D	PP	35	93.1	89.5 (84.2-94.8)	15.4	55.5	78.5	98.5	120.6	
**Visit 1; end of loading dose; day 12 to 21**										
Placebo	ITT	32	91.2	86.3 (81.3-91.3)	13.8	50.1	81.8	95.0	102.5	0.466
Vitamin D	ITT	36	91.6	89.0 (83.3-94.8)	17.0	59.1	73.4	100.2	130.9	
Placebo	PP	29	90.8	85.8 (80.4-91.2)	14.3	50.1	81.2	93.9	102.5	0.468
Vitamin D	PP	35	91.1	88.7 (82.8-94.6)	17.1	59.1	73.0	99.4	130.9	
**Visit 2; end of maintenance dose; week 13 to 16**										
Placebo	ITT	27	87.5	82.7 (76.5-88.9)	15.6	41.2	76.8	93.6	102.8	0.962
Vitamin D	ITT	25	85.3	82.9 (77.3-88.5)	13.6	64.0	71.2	93.4	108.7	
Placebo	PP	23	85.7	81.7 (74.5-88.8)	16.5	41.2	76.6	93.6	102.8	0.651
Vitamin D	PP	18	86.1	83.8 (77.2-90.3)	13.1	64.0	71.4	93.4	108.7	

Abbreviations: ITT, intention-to-treat analysis; Max, maximum; Min, minimum; P25, 25th Percentile; P75, 75th Percentile; PP, per-protocol analysis; SD, standard deviation.

[a] Two-sample two-tailed t-test used to test on difference of the means of two groups. Statistically significant in interim analysis if $p < 0.04$. The p-value of the test was derived by the Satterthwaite method.

Suppl. Table S9. Comparison of the distribution of loading doses hypothetically calculated with the equations of Jansen et al. and von Groningen et al. for the study participants of the VICTORIA trial

Population	Equation	Distribution of loading dose				
		Minimum	25th pct.	Median	75th pct.	Maximum
Total population (n=73)[a]	Jansen	86,302	153,574	199,287	235,521	480,805
	v. Groningen	71,280	133,163	159,310	200,000	388,080
	Δ v. Groningen-Jansen	-4,222	-8,211	-25,207	-15,521	-61,925
	(Δ v. Groningen-Jansen)/Jansen	-5%	-5%	-13%	-7%	-13%
BMI < 30 kg/m² (n=51)	Jansen	86,302	142,822	177,725	212,129	269,981
	v. Groningen	71,280	120,600	141,100	166,160	230,400
	Δ v. Groningen-Jansen	-4,222	-11,062	-20,025	-31,535	-20,381
	(Δ v. Groningen-Jansen)/Jansen	-5%	-8%	-11%	-15%	-8%
BMI ≥ 30 kg/m² (n=22)	Jansen	134,681	215,201	268,210	302,240	480,805
	v. Groningen	120,000	179,200	210,100	249,600	388,080
	Δ v. Groningen-Jansen	5,319	-15,381	-37,110	-26,640	-61,925
	(Δ v. Groningen-Jansen)/Jansen	4%	-7%	-14%	-9%	-13%
25(OH)D < 30 nmol/L (n=49)	Jansen	131,274	192,594	218,503	261,064	480,805
	v. Groningen	110,200	144,624	173,326	211,200	388,080
	Δ v. Groningen-Jansen	-9,474	-34,170	-28,183	-29,264	-61,925
	(Δ v. Groningen-Jansen)/Jansen	-7%	-18%	-13%	-11%	-13%
25(OH)D ≥ 30 nmol/L (n=24)	Jansen	86,302	103,074	140,753	180,252	235,521
	v. Groningen	71,280	96,590	120,300	153,575	225,280
	Δ v. Groningen-Jansen	-4,222	9,261	-3,753	-8,977	15,359
	(Δ v. Groningen-Jansen)/Jansen	-5%	9%	-3%	-5%	7%

Abbreviations: 25(OH)D, 25-hydroxyvitamin D; pct., percentile

[a] One study participant was excluded because he/she had no vitamin D insufficiency at screening and should not have been included in the VICTORIA trial.
